# Tiger attack at a Japanese safari park: a case report

**DOI:** 10.1186/s12245-023-00556-3

**Published:** 2023-11-06

**Authors:** Kouichi Fujii, Jin Kikuchi, Masatoshi Uchida, Masanari Machida, Midori Tsuchiya, Kentaro Hayashi, Nana Maekawa, Hajime Houzumi, Arata Honda, Koji Wake

**Affiliations:** 1 Department of Emergency Medicine, Kamma Memorial Hospital, 2-5 Daikoku-Cho, Nasushiobara, Tochigi, Japan; 2 Department of Emergency and Critical Care Medicine, Dokkyo Medical University, Tochigi, Japan

**Keywords:** Big cat bites, Facial injury, *Pasteurella*, Post-traumatic stress disorder

## Abstract

**Background:**

Big cat bites are highly lethal due to the enormous bite force of these animals. This article reviews the morphology of these types of injuries and key points of management through a survival case at a Japanese safari park.

**Case presentation:**

We report the case of a 26-year-old female keeper who was attacked by a tiger. She was quickly transported to our university hospital by ambulance helicopter. The keeper was severely bitten on the head and face and had wounds all over her body. Craniofacial repair was performed by emergency surgery. She suffered mild facial nerve paralysis and trismus because of being bitten by the tiger and is currently recovering.

**Conclusions:**

A multidisciplinary approach of the severe tiger bites successfully treated a young woman cosmetically and mentally. Animal farms and zoos that keep tigers should take strict measures to avoid direct confrontation with tigers.

## Background

In Japanese zoos and zoological parks, measures are taken to prevent people from directly confronting big cats such as tigers and lions. However, in prior accidents, individuals have been attacked by big cats due to human errors. Big cat bites are highly lethal due to the enormous bite force of these animals [1].

We report on the trauma and psychological management of a young female zookeeper who sustained severe facial trauma and posttraumatic stress disorder from a tiger bite.

### Case presentation

In Japan, 2022 was the year of the tiger. Therefore, many spectators were expected to visit the safari park to see tigers. In the morning just before the gate opened, a 26-year-old female keeper who had worked 2 years there opened the passageway door and was faced with a tiger, which was supposed to have been placed in the cage the previous night. The Bengal tiger was 2 m long and weighed 150 kg. The keeper was attacked from the rear when she momentarily turned her back. First she was bitten on the head, knocked down on the spot, dragged for 3 m, and then bitten several times on her chest and abdomen. She was moved away from the tiger by another keeper and an ambulance was called. After 5 min, the paramedics arrived and provided first aid, such as gauze compression, after which she was directly transported to our university hospital by an ambulance helicopter. Upon presentation, Glasgow Coma Scale was E3V4M6 and respiratory rate was 36/min. Tetanus toxoids 20 IU and immunoglobulins 250 U were administered to prevent infection. Due to persistent bleeding from the head (hemoglobin 10.8 g/dL) and decreased systolic blood pressure to 70 mmHg, an emergency blood transfusion was performed. The patient required 6 units of packed red blood cells and 6 units of fresh frozen plasma. Further, tracheal intubation by rapid sequence intubation was then performed.

**Fig. 1:**
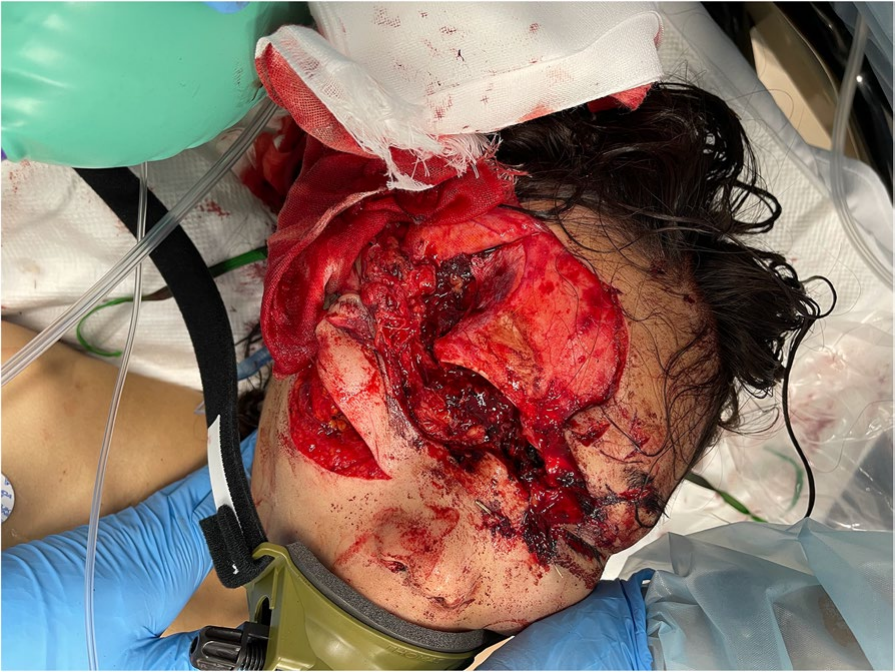
Multiple lacerations in the head and face area

The results of the clinical examination were as follows: Head and face: multiple lacerations on the right midface, both sides of the neck, around the ears, and on both sides of the back of the head due to tiger bites (Fig. 1); the occiput showed a degloving injury on the pericranium, leaving a gap of 5 cm in the center. Chest and back: an open wound measuring approximately 10 cm on the right anterior chest, and the mammary gland had prolapsed; some bite wounds were on the back. Abdomen: an open wound in the lower right abdomen. Extremities: lacerations due to bites on the left thigh and right thumb.

**Fig. 2:**
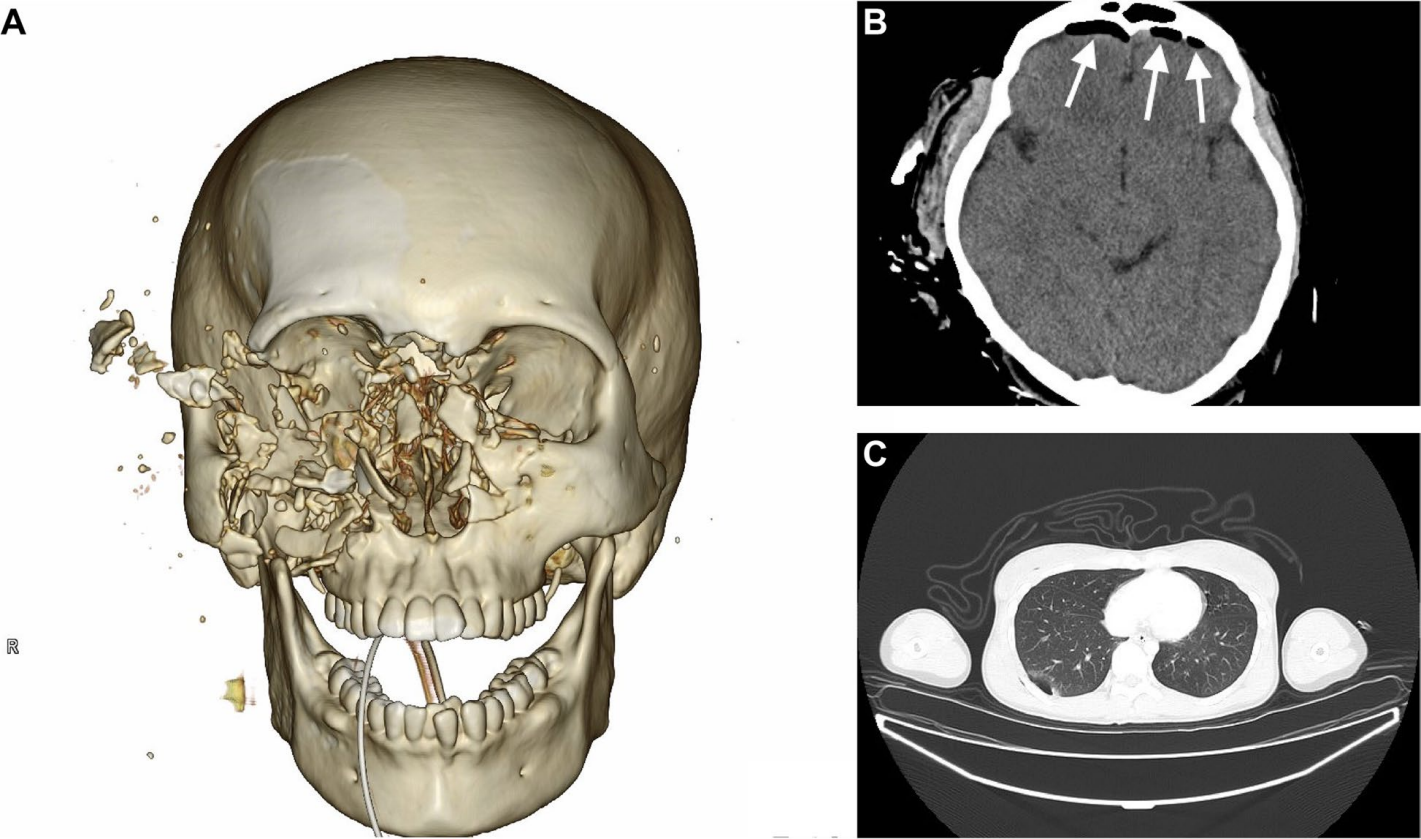
Computed tomography (CT) images. **A** 3D reconstruction of the initial CT scan showing multiple open facial fractures. **B** Head CT scan showing pneumoencephalopathy due to disruption of the frontal sinus (arrow). **C** Chest CT scan showing a small pneumothorax on the right side

**Fig. 3:**
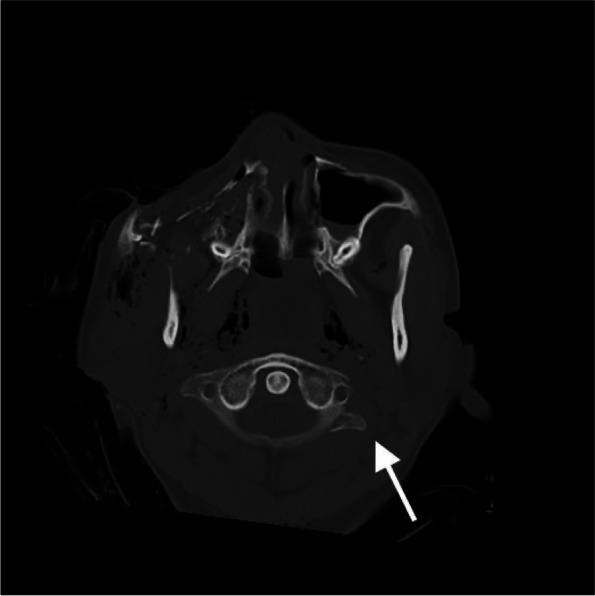
Cervical spine computed tomography scan: C1 cervical transverse fracture (arrow)

**Fig. 4:**
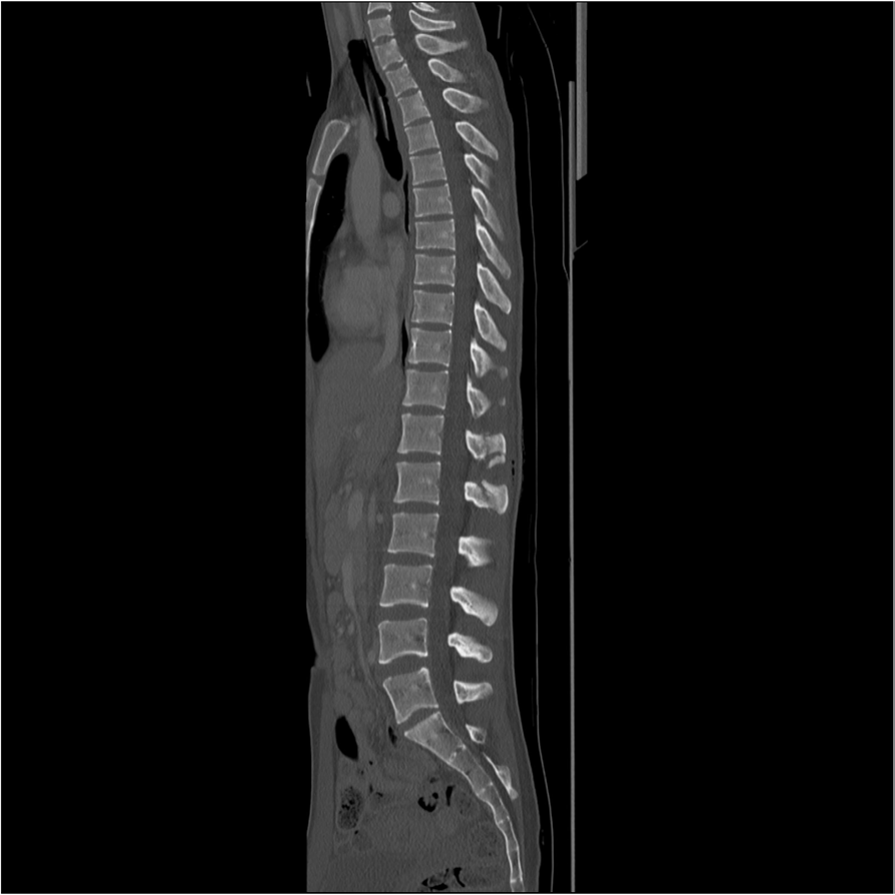
Thoracolumbar spine computed tomography scan: T12 and L1 spinous process fractures

In the initial computed tomography (CT) scan revealed multiple open facial fractures (orbital floor, medial wall fracture, right maxillary comminuted fracture, right zygomatic fracture, nasal bone fracture, anterior skull base fracture, left orbital medial wall fracture, and left maxillary fractures) and pneumoencephalopathy due to the disruption of the frontal and ethmoid sinuses and the entire skull base (Fig. 2A, B). There was a small pneumothorax on the right side (Fig. 2C). A small gas image was detected in the abdominal cavity; however, no organ damage was observed. There were fractures in the C1 cervical transverse process fracture (Fig. 3) and the T12 and L1 spinous processes (Fig. 4). No vascular injury was observed. A 28Fr chest drain was placed in the right thoracic cavity, and a Penrose drain was placed in the abdominal cavity. Wounds found on the chest, abdomen, and extremities were washed and sutured. The initial prophylactic antibiotic was sulbactam/ampicillin for 9 days in the absence of a wound infection.

Emergency surgery was initiated 5 h after arrival at the hospital. A large amount of saline was used to wash the head and face to remove as many fine bone fragments as possible. The prolapsed right eyeball was restored due to optic nerve damage, and the upper and lower bulbar conjunctiva were intact and maintained continuity; however, the eyeball was exposed due to contracture of the right upper eyelid. To maintain the facial shape, only the third largest bone fragment was fixed to a titanium plate. The right zygomatic frontal suture was fixed using a straight 6-hole microplate with a 4-hole 5 mm screw. The suprafrontal suture was fixed with a 4-hole 5 mm screw in a 5-hole Y microplate. The bilateral full-thickness injury of parotid gland and masseter muscle injury were suspected to result in bilateral facial nerve palsy. Skin suturing was performed without soft-tissue repair. No skin defects were observed.

Two Penrose drains were placed in the right anterior chest and one in each of the left lower abdomen, left hip, left groin, and left ilium.

On the day after the surgery, the patient was extubated. Two days after the surgery, enteral nutrition was initiated, and the chest tube was removed. Three days postoperatively, the patient experienced nightmares, underwent psychiatric treatment, and was diagnosed with an acute stress reaction; eszopiclone 3 mg was initiated. Oral intake was initiated on the fifth postoperative day, and the patient was discharged from the intensive care unit.

Fourteen days after the injury, open reduction of the zygomatic fracture was performed. In addition, because the eyelid could not be closed on the right inner canthus side, orbital and nasal bone reconstructions were performed. The patient was doing well and was discharged 21 days after the injury.

**Fig. 5:**
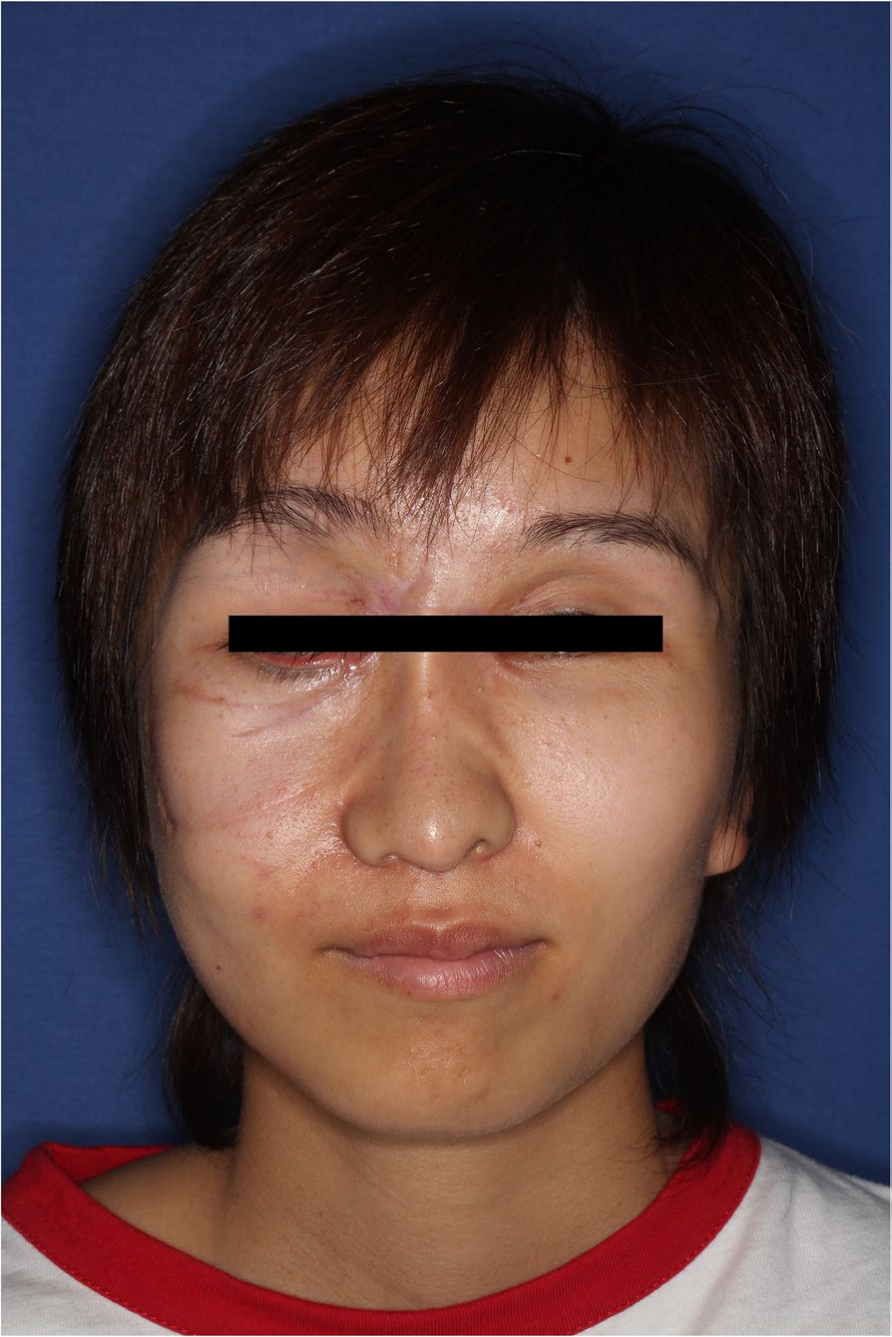
Postoperative result after 7 months show the root of her nose was slightly shifted to the right side

The patient was readmitted to the hospital and underwent surgery for removal of the right mandibular muscle process and right mandibular scar in the Oral Surgery Department because of concomitant contracture-induced trismus. After that, she continued to visit the hospital, and although the root of her nose was slightly shifted to the right side, she regained her cosmetic appearance and her oral intake has improved (Fig. 5).

Five months after being discharged from the hospital, she began complaining of flashbacks and nightmares of the scene at the time of the accident. The patient was diagnosed with posttraumatic stress disorder by a psychiatrist and started on sertraline 50 mg and has been improving since then.

Regarding facial nerve palsy, only the mandibular branch remained on the right side, and only the mandibular branch was completely paralyzed on the left side. Right buccinator muscle branch reconstruction surgery was considered; however, 7 months after being discharged from the hospital, he was able to lift his right eyebrow, and the movement of the orbicularis oris muscle also improved.

## Discussion and conclusions

As wild tigers are declining worldwide, currently, a majority of their attacks on humans are in captivity [2]. Globally, there have been reports of staff being attacked by big cats, such as tigers, at zoos and parks. [3–9]. In Japan, 11 cases of big cat attacks have been reported to date, with 7 deaths. In these zoos, measures have been devised to prevent direct contact with the animals; however, human error has caused accidents in which zookeepers have been attacked [9].

Similar to wild tigers, tigers in captivity instinctively bite the head and neck of the victim, depriving them of movement [5]. Tigers have an extremely strong bite force of 1500 N [10], and there have been cases of death due to damage to the carotid artery caused by being bitten on the neck. Fortunately, this case was not lethal because there were no severe bites on the neck; however, there was a high degree of craniofacial tissue destruction. The patient was transported to a university hospital by helicopter, and emergency surgery was quickly performed by a complex department that mainly focused on plastic surgery. Thorough debridement and irrigation of the affected area and the administration of *Pasteurella*-targeted antibiotics and tetanus toxoids resulted in effective control without wound infection during the course of hospitalization.

Due to the severe facial damage, multiple functional disorders such as visual impairment due to left optic nerve rupture, bilateral facial paralysis due to facial muscle damage, and trismus due to contracture occurred, and multiple surgeries were performed. Therefore, long-term follow up is still required. Aside from the craniofacial lesion, the thoracic injury extended into the thoracic cavity and formed a pneumothorax. Multiple lacerations and fractures were also observed. As tiger canine teeth are long, bites and claw injuries can affect the entire body; therefore, detailed whole-body trauma assessment is important. CT scan has been reported to be useful for a detailed whole-body search, and it was extremely effective in this case [7].

The patient in this case was a young woman, and cosmetic considerations were very important to improve her aesthetics. In such cases, mental shock may occur and lead to an acute stress reaction and posttraumatic stress disorder; therefore, a psychiatric approach is also very important. Multidisciplinary treatment including many departments such as plastic surgery, otolaryngology, surgery, and psychiatry was effective. Therefore, treatment at a large hospital such as our university hospital is desirable, and early or direct transportation should be considered [11].

Tiger bite, such as this case, causes severe trauma to the whole body, especially the head and neck region. In this case, a multidisciplinary approach including plastic surgery, otolaryngology, surgery, and psychiatry at the university hospital successfully treated a young woman cosmetically and mentally. Animal farms and zoos that keep tigers should take strict measures to eliminate human error and avoid direct confrontation with tigers.

## Data Availability

Not applicable.

## Abbreviation

CT: Computed tomography

## References

[1] Szleszkowski Ł, Tannhäuser A, Jurek T (2017). Compound mechanism of fatal neck injury: a case report of a tiger attack in a zoo. Forensic Sci Int.

[2] Kelly A, Goosen J, Venter M, Younus A (2020). Management of Bengal tiger attacks – a case report and literature review. Interdiscip Neurosurg.

[3] Kohout MP, Percy J, Sears W, Yeo JD (1989). Tiger mauling: fatal spinal injury. Aust N Z JSurg.

[4] Cohle SD, Harlan CW, Harlan G (1990). Fatal big cat attacks. Am J Forensic Med Pathol.

[5] Wiens MB, Harrison PB (1996). Big cat attack: a case study. J Trauma.

[6] Schiller HJ, Cullinane DC, Sawyer MD, Zietlow SP (2007). Captive tiger attack: case report and review of the literature. Am Surg.

[7] Anderson M, Utter P, Szatkowski J, Patrick T, Duncan W, Turner N (2008). Cervical spine injury: tiger attack. Orthopedics..

[8] Hejna P (2010). A fatal leopard attack. J Forensic Sci.

[9] Tantius B, Wittschieber D, Schmidt S, Rothschild MA, Banaschak S (2016). Two fatal tiger attacks in zoos. Int J Legal Med.

[10] Christiansen P, Wroe S (2007). Bite forces and evolutionary adaptations to feeding ecology in carnivores. Ecology.

[11] Dabdoub CF, Daubdoub CB, Chavez M, Molina F (2013). Survival of child after lion attack. Surg Neurol Int.

